# Identification and molecular functional analysis of genes associated with addiction using integrated genetic web-based programs and databases

**DOI:** 10.3389/fgene.2026.1763510

**Published:** 2026-05-22

**Authors:** Waheeda A. Hossain, Merlin G. Butler

**Affiliations:** Departments of Psychiatry and Behavioral Sciences and Pediatrics, University of Kansas Medical Center, Kansas City, KS, United States

**Keywords:** addiction and alcoholism genes, addiction and related behavior, biological processes, genetic mechanisms, molecular functions, pathways and gene–disease associations, STRING and GeneAnalytics integrated web-based programs, databases and categories

## Abstract

**Background:**

Addiction is a chronic, relapsing neuropsychiatric disorder despite adverse consequences. Addiction involves neurobiological changes in the reward, motivation, and memory systems, particularly affecting the dopaminergic pathways due to a complex interaction between inherited traits and life experiences.

**Methods:**

To explore the role of genes in addictive behavior, we compiled a list of 332 clinically relevant genes from literature sources and searched GeneAnalytics, STRING web-based programs, and integrated genetic and protein databases for interactions and molecular profiles of genes in tissues and cells, diseases, pathways, biological processes, cellular components, functions, phenotypes, and compounds.

**Results:**

We identified 332 genes associated with addictive behavior and predominantly expressed in the brain associated with schizophrenia, nervous system disorders, and cancer. Nine of the top 10 related pathways showed GPCR protein interaction and signaling involving the *CREB1*, *MAPK1*, *MAPK3*, and *AKT1* genes. The *BDNF* gene was recognized in eight of the top 10 high-scoring phenotypes involving the neurotransmitters dopamine and glutamate. We chose to study *AKT1* as the most identified gene when searching web-based programs and integrated genetic and protein databases. This gene encodes protein kinases that are required for protein phosphorylation, fundamental for most cellular functions, stabilization, regulation of targeted proteins including cell cycle control and signal transduction, neuron formation, and neurological function including the hippocampus. We identified the important PI3/AKT/mTOR signaling pathway that integrates extracellular growth signals, promotes proliferation, and inhibits apoptosis, which is important for brain function. These genes also overlap with other genes associated with ATPase activity, metabolism, and chaperone protein functions that play a role in addiction. In addition, we identified 29 genes that are shared with alcoholism/alcohol use disorder and overlap with interactive molecular functions and processes between alcoholism and addiction.

**Conclusion:**

Our study identified 332 addiction genes from literature sources and used *in silico* integrated genetic approaches with two searchable web-based programs to provide insights into the complex molecular and genetic architecture of addiction. We identified cell cycle control factors, extracellular growth, signal transduction, and metabolism. The pathways involved selective protein phosphorylation and transduction of proteins as well as neurotrophic factors with mechanisms impacting nervous system development, plasticity and function when disturbed, leading to addictive behavior. Several genes associated with addiction overlapped with those associated with alcohol use disorder.

## 1 Introduction

Addiction is a chronic, relapsing neuropsychological disorder characterized by compulsive drug-seeking, continued use despite harmful consequences, and long-lasting changes in the brain (Volkow et al., 2016; NIDA, 2020). Addiction involves a loss of control over behavior or substance use and is often associated with physical or psychological dependence on the substance or behavior influenced by genetic factors. Addictive behaviors and substance use disorder including alcohol consumption, tobacco smoking, and unsanctioned psychoactive drug (e.g., cocaine and opioids) use are major contributors to the global burden of morbidity and premature death (Lim et al., 2012). These behaviors are often driven by alterations in neural circuitry that regulate impulse control, decision-making, and reward processing. The prevalence and co-occurrence of 11 types of addictions investigated by Sussman et al. (2011); Sussman et al. (2014) included 90% of addictions listed for alcohol, tobacco, illicit drugs (including marijuana), gambling, eating/food, internet, love, sex, exercise, work, and shopping. Addiction can also be related to substances (e.g., alcohol, drugs, or nicotine) or behaviors (e.g., gambling, internet use, sex, or shopping). Approximately one-half of the US adult population shows signs of 11 types of addiction, as discussed by Sussman et al. (2011). Co-occurrence of addictions is reported at 23 percent on average. Substantial variability of co-occurrences is also noted among addictions with a 10%–50% overlap (Luczak et al., 2017).

Addiction is often seen solely as a result of poor choices or environmental influences. Research has shown that genetics play a significant role in shaping an individual’s susceptibility to addictive behaviors ranging from alcohol dependency to gambling and substance abuse. Addictive behavior is increasingly understood as a complex interaction between inherited traits and life experiences which arise from multiple pathways and manifest themselves in innumerable ways. Genetic and environmental risk factors account for roughly half of an individual’s risk for developing an addiction (Nestler, 2013). Many genes implicated in addiction have an estimated range of 40%–60% heritability along with the involvement of genetic risk loci or recognized genetic susceptibility (Goldman et al., 2005; Hiroi and Agatsuma, 2005; Hatoum et al., 2023).

To understand the role of genetics, we searched the literature and analyzed genetic and protein web-based programs with databases using identified genes and proteins involved in addiction for molecular and genetic profiling. We hypothesize that the functional impact of the genes influencing addiction will converge on the biological processes, molecular mechanisms, pathways, and associated diseases addressed in our report.

## 2 Materials and methods

### 2.1 Searchable literature and web-based programs and databases

#### 2.1.1 Literature and websites queried

We focused on addiction and addictive behavior in humans by systematically identifying, collecting, and analyzing genetic data from literature and web-based sources. We undertook a structured multistep approach as reported in previous comparable studies to identify causative and implicated genes with their role in protein production and function related to human diseases. We followed the approach and methods used in other peer-reviewed publications such as alcoholism or alcohol use disorder (Manzardo et al., 2014; 2015), obesity (Butler et al., 2015a; Gabrielli et al., 2017), autism (Butler et al., 2015b; Gabrielli et al., 2019), infertility (Butler et al., 2015c), schizophrenia (Khanzada et al., 2017; Sundararajan et al., 2018), and bipolar disorder (Douglas et al., 2016). First, comprehensive literature sources and disease-specific databases were searched, including PubMed (https://www.ncbi.nlm.nih.gov/pubmed) and other computer web-based resources, using key words focused on addiction, addictive behavior, genes, gene variants, mutations, and expression in humans; for example, we searched “addiction and genes,” “addictive behavior and genes,” etc., from literature or other sources. We identified clinical, experimental, structural, and/or functional genetic findings including genome-wide association studies (GWAS) and other studies based on specific genes recognized from literature and other sources that cause or are related to addiction or addictive behavior. Often, gene names or symbols were found when searching for keywords related to addiction in literature or other sources in the title, abstract, keyword section, or body of the publication. The source would then be reviewed to identify the content and evidence of the gene or genetic findings playing a role in addiction. Second, curated and authoritative genetic databases were searched to ensure additional clinical relevance and strength of evidence by identifying more sources representing a specific gene found and its role in addiction. These sources included the National Center for Biotechnology Information (NCBI) (http://www.ncbi.nlm.nih.gov/), Online Mendelian Inheritance in Man (OMIM) (www.OMIM.org), GeneCards (https://www.genecards.org), and GeneReviews (https://www.genereviews.org). Where available, specialized gene catalogs or sources that focus on specific diseases or disorders were included in the identification and analysis of genes for addiction. Third, the inclusion criteria were documented to support specific gene involvement, such as identifying replicated studies such as specific gene variants, mutations, single-nucleotide polymorphisms, GWAS, whole-exome/genome sequencing, microarray gene expression and/or copy number analyses, family-based linkage studies, and functional gene expression profiling in relevant human tissues (e.g., brain regions) implicated in reward and impulse control, for the inclusion of the gene. Importantly, the DISGENET (www.disgenet.com) website, which is a large repository for disease-specific human genes, was also searched as a major investigative source to further add and confirm that genes found in our literature and other sources selected for the study on addiction or addictive behavior were in agreement prior to inclusion. Hence, all the identified genes were analyzed or validated with evidence of causation or contribution to behavioral and neurological phenotypes associated with addiction before including them in our study. Our structured strategy to search for genes associated with addiction or addictive behavior using multiple sources led to an assembly of a comprehensive, evidence-based gene list that is relevant to addiction pathology while maintaining scientific rigor and reproducibility. Finally, we compiled a tabular list of 332 clinically relevant genes in humans based on a research undertaken in the year 2025.

Our comprehensive list of genes associated with addiction and their role showed key components involving the neurotransmitter system, which appears to be central to addiction vulnerability, including dopaminergic signaling (*DRD2*, *COMT*, and *MAOA*), serotonergic pathways (*HTR2A* and *TPH2*), GABAergic and glutamatergic neurotransmission (*GABRA2*, *GABBR1*, *GRIN2B*, and *GRM5*), and opioid receptor signaling (*OPRM1*, *OPRD1*, and *OPRK1*). Other genes involved reward-related brain plasticity and stress response, such as *BDNF* (brain-derived neurotrophic factor), *CREB1* (cAMP response element-binding protein 1), *CRH*, *NR3C1*, and *FOSB*. These genes play important roles in reinforcement learning, craving, and relapse. In addition, metabolic genes that affect how substances are processed in the body (*CYP2A6*, *CYP2D6*, *ADH1B*, and *ALDH2*) were identified as having a potential role in substance response, toxicity, and affecting treatment strategies (e.g., OMIM and GeneCards). Overall, our compiled gene set reflected a system-level view of addiction by bringing together key processes that include neurotransmission, intracellular signaling pathways (such as MAPK, PI3K-AKT, and mTOR), neuroinflammation, synaptic plasticity, and substance metabolism involving biological processes in the development and progression of addictive disorders (Koob et al., 1998; Nestler, 2000; Liu et al., 2006; Volkow et al., 2012; Volkow and Boyle, 2018; Uhl et al., 2019; Maldonado et al., 2021; Agrawal et al., 2025).

The major list of 332 genes for addiction was then focused upon in our computational web-based integrated analysis using two separate computer programs and databases. First, the searchable GeneAnalytics program was used to generate an overall analytical appraisal of the identified gene set of 332 addiction and addictive behavior-related genes to determine their involvement in tissues and cells, diseases, super pathways/pathways, biological processes, molecular functions, phenotypes, and compounds. We followed the protocol implemented in previous studies on autism, obesity, and psychiatric disorders (Gabrielli et al., 2017; 2019; Khanzada et al., 2017; Sundararajan et al., 2018). Second, the searchable STRING web-based program and database were used to analyze genes individually, and their encoded proteins. Important gene(s) were identified using the GeneAnalytics approach to recognize and characterize their protein–protein interactions, biological processes, molecular functions, pathways, and disease–gene associations (Szklarczyk et al., 2011; 2023; Barakat and Butler, 2024; Genovese and Butler, 2025; Butler et al., 2025; Butler, 2026).

### 2.2 Searchable web-based programs and databases queried

#### 2.2.1 GeneAnalytics

The master list of identified addictive behavior-associated genes was then analyzed using the GeneAnalytics (http://geneanalytics.genecards.org/) program, as reported previously by our research group for schizophrenia, autism, and obesity (e.g., Gabrielli et al., 2017; 2019; Khanzada et al., 2017). This established searchable program was developed by LifeMap Sciences as part of the GeneCards Suite (http://www.lifemapsc.com/products/genecards-suite-premium-tools/). It is used as a comprehensive, easy-to-apply gene-set analysis tool to contextualize the expression patterns and functional signatures embedded in advanced genomic expression, sequencing, and microarray datasets (Ben-Ari Fuchs et al., 2016). Other included databases were MalaCards (http://www.malacards.org/)—human disease database and PathCards (http://pathcards.genecards.org/)—biological pathways database and expression-based analyses including manually curated expression data from normal and diseased tissues to study the gene–tissue association relationships.

The GeneAnalytics program analyzed the aggregate addictive behavior gene set cross-referenced against various categories such as tissues and cells, diseases, pathways, gene ontology (GO)–biological processes, GO–molecular functions, phenotypes, and compounds following established protocols (Butler et al., 2015; Ben-Ari Fuchs et al., 2016; Butler et al., 2016; Gabrielli et al., 2017; Khanzada et al., 2017). Scores were assigned by the GeneAnalytics program analysis for each of the above categorized fields using an algorithm native to GeneAnalytics which investigates the number of “matched” genes (between our master list and those found in GeneCards) and the total number of genes identified for a particular category. Scoring fields were generated by using an algorithm based on their relevance to the gene set further subdivided and assigned into three categories, namely, “high-score matches,” “medium-score matches,” and “low-score matches,” as noted in the description prior to each category table listed below. As an example, for the “tissues and cells” category listed below, each gene and entity has a score that is based on the entity type and the gene annotations based on information from the scientific literature searched and captured by the GeneAnalytics program, with bioinformatics and calculations performed using expression data from the Life Map Discovery database. The entity scores that are generated and classified are rated as high, medium, or low based on their quality. Matching genes from addictive behavior-associated disease categories were then cross-checked against the pathway category, and corresponding frequencies were recorded, including the number of times each gene appeared in a separate pathway. The GeneAnalytics program develops an overall analytical statistically derived score listed as a numerical value that reflects how strongly an input collective gene list is associated and, therefore, grouped into seven biological categories. These include the following: 1) Tissues and Cells, 2) Diseases, 3) Superpaths/pathways, 4) Gene Ontology (GO)–Biological processes, 5) GO–Molecular functions, 6) Phenotypes, and 7) Compounds. Further descriptions are listed below.

##### 2.2.1.1 GeneAnalytics categories

###### 2.2.1.1.1 Category 1 (Tissues and Cells)

Tissues and cells were analyzed representing the expression of genes in the query set, which allows for understanding tissue-specific functions or cell-type involvement. Information on normal cells, anatomical compartments, organs, and tissues was matched to the query gene lists as specific entities. The GeneAnalytics program (https://geneanalytics.genecards.org/features/tissues-cells/) uses data from the updated LifeMap Discovery database (http://discovery.lifemapsc.com) (Edgar et al., 2013).

###### 2.2.1.1.2 Category 2 (Diseases)

This category links diseases to the genes for the known diseases and indicates potential roles in disease development or progression. It utilizes the MalaCards database (http://www.malacards.org) (Rappaport et al., 2013) and GeneCards sources to analyze gene–disease relationships.

###### 2.2.1.1.3 Category 3 (Superpaths/Pathways)

Superpaths/Pathways include the groups of genes based on their participation in shared biological pathways and provide insights into broader cellular processes and networks. This program uses PathCards to unify multiple pathway sources available in GeneCards in order to form “superpaths.” The matching algorithm used is based on the GeneDecks set distiller tool (Stelzer et al., 2009) and normalized genetic influences in matching score-based information and cumulative binomial distribution. Significant results have a *p-*value <1/total number of potential matches in the category. The scores are equal to the −log_2_ of the resulting *p-*value.

###### 2.2.1.1.4 Category 4 [Gene Ontology (GO)–Biological Processes]

GO–biological processes categorize genes according to their involvement in the processes, such as development, metabolism, or cellular communication. Biological processes are then integrated and based on the GeneCards database from the Gene Ontology Project (www. geneontology.org). According to the consortium description, the biological process is a series of events accomplished by one or more organized assembly of molecular functions that are helpful for gaining an understanding of these related events.

###### 2.2.1.1.5 Category 5 (GO–Molecular Functions)

GO–molecular functions classify genes based on their functions such as protein binding or enzymatic activity. A molecular function is defined as the elemental activity of a gene product at the molecular level that is important for understanding the biology of the set of genes and their combined functions (http://geneontology.org/page/ontology-documentation).

###### 2.2.1.1.6 Category 6 (Phenotypes)

Phenotypes refer to the association of genes and their observed phenotypic characteristics that are useful for understanding the potential consequences of gene alterations. The phenotypes section of the GeneCards database describes the associations between genes and observable traits, which helps understand the biological and clinical significance of gene alterations. It incorporates data information from Mouse Genome Informatics (http://www.informatics.jax.org/) and Human Phenotype Ontology (http://human-phenotype-ontology.github.io/).

###### 2.2.1.1.7 Category 7 (Compounds)

Compounds identified are related to potential interactions between the genes and specific chemical compounds, including drugs or other molecules, whereby information about the role of chemicals will be helpful in addressing concerns raised for the treatment of addictions. The GeneCards database integrates information from various sources for compound and drug associations, including DrugBank (https://www.drugbank.ca/), ApexBio (http://www.apexbt.com/), Drug Gene Interaction Database (http://dgidb.genome.wustl.edu/), FDA-Approved Drugs (http://www.accessdata.fda.gov/scripts/cder/drugsatfda/index.cfm), ClinicalTrials.gov (https://www.clinicaltrials.gov/), PharmGKB (https://www.pharmgkb.org/), International Union of Basic and Clinical Pharmacology (http://www.guidetopharmacology.org/), Novo seek, Human Metabolome Database (http://www.hmdb.ca/), BitterDB (http://bitterdb.agri.huji.ac.il/dbbitter.php), and Tocris Biosciences (https://www.tocris.com/).

#### 2.2.2 STRING program and database

The STRING web-based program and database (www.string-db.org) were used to further analyze protein–protein interactions for our genes and the encoded protein study in addiction. STRING is an established searchable program for the identification of predicted protein–protein interactions, genetic mechanisms, and functions with associated disease pathology (Szklarczyk et al., 2023; 2025). The protein interactions may be both direct (physical) and indirect (functional), but they are derived from genomic context predictions, high-throughput laboratory experiments, and automated text mining from literature sources with other databases and include conserved co-expression patterns for genes. The STRING program provides a statistical approach by analyzing individual genes and their encoded proteins.

STRING uses four separate criteria for analysis including the Count in network (CIN) function, which indicates how many proteins are present in the visualized network. It is annotated with a particular term and out of how many proteins in total are found with this assigned term. Strength describes the size of the enrichment effect and is assigned as a ratio of the number of term-annotated proteins found in a visualized network related to the number of proteins expected to be annotated in a random network of the same size (log10 (observed/expected)). The false discovery rate (FDR) is calculated to examine the significance of enrichment with a statistical measurement to conceptualize the rate of type-I (false positive) errors in the null hypothesis testing. The FDR is reported as *p*-values corrected for multiple testing within each category. The signal is defined as a weighted harmonic mean between the observed/expected ratio and -log (FDR). It is meant to balance the metrics of larger and smaller terms for more intuitive ordering of the enriched terms. This searchable *in silico* web-based program has been used by our research group (Genovese and Butler, 2025) and others, with hundreds of published reports in the literature.

## 3 Results

### 3.1 GeneAnalytics data analysis

Based on our study, a total of 332 genes associated with addictive behaviors were identified in the literature and other sources. These genes code for proteins and functions that represent a broad range of clinically relevant and known addictive behavior-related genes, forming the foundation of a master list of genes linked to human addictive behavior. The updated list of 332 clinically related genes for addictive behavior is shown in Table 1. Unsurprisingly, some genes were found to relate to other disorders/diseases besides addiction. For example, FTO and *POMC* genes are important recognized genes for obesity (Butler et al., 2015; Butler et al., 2016), while the *MAOA*, *MAOB*, *CLOCK*, *PTEN*, *GABBR2*, *PRD4*, and *COMT* genes are recognized to play a role in behavior, autism, and psychosis (Butler et al., 2015; Khanzada et al., 2017; Sundararajan et al., 2018; Gabrielli et al., 2019; Butler, 2024; Genovese and Butler, 2025).

**TABLE 1:** Updated list of 332 clinically relevant and known genes for addictive behavior arranged in an alphabetical order and analyzed by the GeneAnalytics program.

ABCB1	CCL11	DUSP1	GRIN2B	KEAP1	MYD88	PNO1	SLC6A4
ABL1	CD200R1	DUSP2	GRIN3A	KIFC1	MYH6	PNOC	SLCO6A1
ACE	CD46	E2F1	**GRM1**	**KIT**	MYO6	POLQ	SMR3B
ACHE	CD8A	EGFR	**GRM2**	KLF6	NBEAL1	POMC	SNAP25
**ACTB**	**CDK5**	EHMT1	GRM3	KMO	NCK2	POU3F2	SOX2
ADCYAP1R1	CDK7	EIF4E	GRM5	KMT2A	NFASC	PPP3CA	SRRM2
ADH1B	CDK9	EPHA3	**GSN**	KRAS	NFE2L2	PRS	ST11
ADRA2A	CECR	EPHB2	GSTK1	KRIT1	NFE2L3	PSMG1	STAT1
AGRP	CFH	EPO	GSTT2	KRT7	NKTR	PTBP1	STAT3
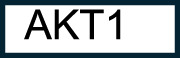	**CHN2**	ERBB2	GSTT2B	LIG3	NPY	PTEN	SUGP1
ALDH2	CHRM2	ERBB3	H2AX	LIMK1	NR2E1	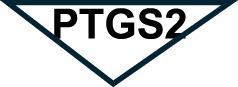	SYT1
ALK	CHRM5	ERVK-18	HCRT	LINC02210-CRHR1 readthrough	NR3C1	PTP4A3	TAAR1
ALOX5	**CHRNA3**	ERVK-20	**HCRTR1**	MAK16	NR3C2	RAF1	TAC1
ANGPT1	**CHRNA4**	ERVK-32	HCRTR2	MAOA	NR4A2	RBFOX1	TACR1
ANKK1	**CHRNA5**	ERVW-1	HDAC6	MAOB	NRAS	RBMS3	TAFAZZIN
ANKS1B	**CHRNB4**	ESR1	HIC1	MAP2K7	NRCAM	RBP4	TAL1
AR	CLMP	FAAH	HJURP	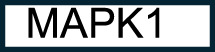	NRXN3	RENBP	TBK1
ARHGEF1	CLOCK	**FGF13**	HMGB1	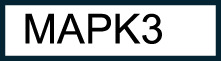	NTRK2	RGS20	TBX1
ARRB2	CNIH3	FGF2	HOMER1	MAPK7	OCA2	ROBO1	TFAP2C
ARSI	**CNR1**	FGFR2	HSF1	MEIS1	OPN1LW	RUNX1	TH
ARTN	COMT	FHIT	HSP90AA1	MET	OPRD1	RXRA	THAS
ATG5	COPD	FHP1	HSP90B2P	MIR10B	OPRK1	SALL4	TKT
ATG7	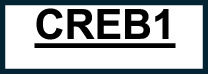	FIP1L1	**HTR2A**	MIR126	OPRL1	SAMMSON	TLR4
ATOH1	CREBBP	**FN1**	HTR2C	MIR134	OPRM1	SAT1	TLX1
**ATP6V1B2**	**CRH**	FOSB	**IGF1**	MIR139	OR2AG1	SCD	TLX3
AVP	CRHBP	FOXF1	IGF1R	MIR21	OSR1	SCLY	TP53
BAG3	**CRHR1**	FTO	IGF2	MIR29C	OXA1L	SEC23B	TPH2
BAS	CSF3	G6PD	**IGFBP2**	MIR331	PAG1	SERPINF2	TPSG1
BCHE	CSMD1	**GABBR1**	IGFBP7	MIR495	PCBP4	SIGMAR1	TRPV1
BCL2	CSN1S1	**GABBR2**	IL11	MIRLET7D	PCYT1A	SLC16A3	TSPO
**BDNF**	CSTA	GABPA	IL15	MITF	PDE4A	SLC16A4	TTC21B
BMI1	CYP19A1	GABRA2	IL1B	MLH1	PDLIM7	SLC18A2	TXN
BRAF	CYP2A6	**GAL**	IL5	MLXIPL	PDYN	SLC1A2	ULK3
C1QBP	CYP2D6	GCG	ING2	MMP1	PEA15	SLC1A5	VPS13A
C1QL1	CYP3A4	**GDNF**	INSR	MMP9	PEBP1	SLC2A1	YAP1
CALM1	DBH	GGT1	IRF4	MST1R	PIK3CA	SLC2A12	ZGLP1
CALM2	DNER	GGT5	IRF6	MT-CO2	PIK3CB	SLC2A3	ZHX2
CALM3	**DRD1**	**GHSR**	**JAG1**	MTCO2P12	PIK3CD	SLC2A8	ZNF804A
CAMK4	**DRD2**	GLP1R	**KCNJ6**	MTHFR	PIK3CG	SLC4A7	​
CAMKMT	**DRD3**	**GLS**	KCNJ9	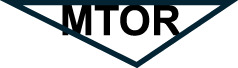	PITX2	SLC6A1	​
CARM1	DRD4	**GPR88**	KCNQ4	MYB	PLCG1	SLC6A2	​
**CARTPT**	DSTN	GRIN2A	KDM3A	MYC	PLK1	SLC6A3	​

Thirty-six genes showed an overlap in separate GeneAnalytics analyses located into categories. They were found in all five high-matched anatomical brain compartments in the Tissues and Cells category described, which are shown in bold and are underlined. Genes that were found in all top 10 diseases in the Disease category are represented with a triangle. Genes that were present in nine of the top 10 pathways in the Superpaths category are represented with a rectangle.

### 3.2 Description of the GeneAnalytics web-based categories and results

#### 3.2.1 Tissues and cells

The study of tissues and cells showed that the 332 genes are matched with 1,528 entities, and, of these, 1,048 were matched to the *in vivo* group and 480 were matched to the *in vitro* group*.* The matched entities represented 807 cells, 135 anatomical compartments, 36 organs/tissues, and 550 from high-throughput experiments. One anatomic brain compartment, the cerebellum, was qualified as a high match with a rating score of 15.40 (see Table 2). Ten medium-score matches (range: 13.20 to 7.27) were found, with 1,517 genes showing low-score matches (range less than 6.36). A total of 36 genes were found to overlap in all five high-matched anatomic brain compartments, which are represented as bold and underlined in Table 1.

**TABLE 2:** Top five tissues and cells with the expression of clinically relevant and known genes for addictive behavior.

GeneAnalytics score	Tissues and cells	Total number of genes that were expressed in tissues and cells identified from integrated databases	Number of matched addictive behavior-associated genes from the master list (%)	Number of matched genes from integrated databases (%)	The 67 overlapping genes in the top three tissues and cells
15.40	Cerebellum	3,335	115/332 (34.6)	115/3,335 (3.4)	ACHE, **ACTB** , ADRA2A, ATP6V1B2, **BDNF** , CARTPT, CDK5, CHN2, CHRNA3, CHRNA4, CHRNA5, CHRNB4, CNR1, **CREB1** , CRH, CRHR1, DRD1, DRD2, DRD3, DUSP1, FGF13, FN1, GABBR1, GABBR2, GAL, GDNF, GHSR, GLS, GPR88, GRM1, GRM2, GSN, HCRTR1, HOMER1, HTR2A, **IGF1** , IGFBP2, JAG1, KCNJ6, KIT, LIMK1, MAOB, **MAPK1** , MET, MST1R, NCK2, **NPY** , NR3C1, NRXN3, OPRK1, PDYN, PITX2, PNOC, POMC, PPP3CA, SLC1A2, SLC6A1, SLC6A2, SLC6A3, SLC6A4, SNAP25, SOX2, STAT3, SYT1, TAC1, **TH** , and TKT
13.20	Medulla oblongata	2,179	107/332 (32.2)	107/2,179 (4.9)	
12.42	Cerebral cortex	5,055	142/332 (42.7)	142/5,055 (2.8)	
12.32	Hypothalamus	1,736	95/332 (28.6)	95/1,736 (5.5)	
11.16	Thalamus	1,666	86/332 (25.9)	86/1,666 (5.2)	

Colored genes represent those present in more than one analyzed category.

#### 3.2.2 Diseases

The top 10 high-scoring associated diseases were found using the compiled list of 332 Addictive Behavior genes and the GeneAnalytics program with integrated genomic databases. There were 317 matched diseases of the 5,004 disease entities. There were 226 high-score matches (range: 73.62 to 12.93), 512 medium-score matches (range: 12.86 to 6.40), and 4,266 low-score matches (range: 6.39 to 0.77). Schizophrenia had the highest score (score: 73.62), closely followed by nervous system diseases (score: 71.36) and colorectal cancer (score: 66.71). The percentage of the matched genes in each disease category is given in Table 3. The *MTOR* and *PTGS2* genes were found in all top 10 diseases in the Disease category (see Table 1, where they are represented with a triangle).

**TABLE 3:** Top 10 diseases associated with clinically relevant and known genes for addictive behavior.

GeneAnalytics score	Disease	Total number of genes identified in diseases from integrated databases	Number of matched addictive behavior-associated genes from the master list (%)	Number of matched genes from integrated databases (%)	The 18 overlapping genes in the top three diseases
73.62	Schizophrenia	523	93/332 (28.0)	93/523 (17.8)	ABCB1, **ACTB** , **AKT1** , **CREB1** , CYP3A4, ESR1, GRIN2A, **IGF1** , IL1B, MIR29C, MMP9, MTHFR, MTOR, **NPY** , PTGS2, SLC2A1, TAC1, and TP53
71.36	Nervous system disease	950	102/332 (30.7)	102/950 (10.7)	
66.71	Colorectal cancer	1,617	93/332 (28.0)	93/1,617 (5.7)	
63.22	Lung cancer	1,347	79/332 (23.8)	79/1,347 (5.9)	
62.67	Breast cancer	1,564	83/332 (25.0)	83/1,564 (5.3)	
57.10	Body mass index quantitative trait locus 11	881	79/332 (23.8)	79/881 (9.0)	
55.90	Alzheimer disease, familial, 1	901	83/332 (25.0)	83/901 (9.2)	
53.10	Type 2 diabetes mellitus	623	72/332 (21.6)	72/623 (11.5)	
49.83	Prostate cancer	1,223	68/332 (20.4)	68/1,223 (5.6)	
47.02	Diabetes mellitus	584	64/332 (19.2)	64/584 (10.9)	

Colored genes represent those present in more than one analyzed category.

#### 3.2.3 Superpaths/Pathways

Of the identified 332 genes for addictive behavior, 281 genes matched to 487 Superpaths. There were 384 high-scoring entities (range: 160.09 to 13.31), 103 medium-score matches (range: 13.28 to 10.24), and no low-score matches. The top 10 Superpaths and the number of matched genes are shown in Table 4. Signal transduction, endometrial cancer, and G protein-coupled receptor (GPCR) downstream signaling across chemical synapses included the top three pathways in the Superpaths category, with matching scores of 160.09, 107.95, and 101.13, respectively. The CREB1, MAPK1, MAPK3, and AKT1 genes were present in nine of the top 10 pathways in the Superpaths category (see Table 1, where they are represented with a rectangle).

**TABLE 4:** Top 10 superpaths associated with clinically relevant and known genes for addictive behavior.

GeneAnalytics score	Superpaths	Total number of genes identified in Superpaths from integrated databases	Number of matched addictive behavior-associated genes from the master list (%)	Number of matched genes from integrated databases (%)	The 12 overlapping genes in the top three superpaths
162.03	Signal transduction	2,579	139/332 (41.8)	139/2,579 (5.4)	**AKT1** , CALM1, CALM2, CALM3, **CREB1** , **EGFR** , GRM3, KRAS, **MAPK1, MAPK3** , NRAS, and PIK3CA
108.49	Endometrial cancer	201	40/332 (12.0)	40/201 (20.0)	
102.13	GPCR downstream signaling	708	62/332 (18.6)	62/708 (8.7)	
87.54	MIF-mediated glucocorticoid regulation	681	56/332 (16.8)	56/681 (8.2)	
86.69	Prolactin signaling	432	46/332 (13.8)	46/432 (10.6)	
78.67	Signaling by receptor tyrosine kinases	521	47/332 (14.1)	47/521 (9.0)	
77.77	CREB pathway	529	47/332 (14.1)	47/529 (8.9)	
77.16	Malignant pleural mesothelioma	409	42/332 (12.6)	42/409 (10.3)	
73.29	RAF/MAP kinase cascade	571	47/332 (14.1)	47/571 (8.2)	
71.65	Methylphenidate pathway, pharmacodynamics	18	15/332 (4.5)	15/18 (83.3)	

Colored genes represent those present in more than one analyzed category.

#### 3.2.4 GO–biological processes and GO–molecular functions

From the 332 addictive behavior-associated genes, 270 genes were matched to 291 GO–biological processes with top 10 high-scoring entities. There were 226 high-score matches (range: 90.43 to 13.31), 65 medium-score matches (range: 13.25 to 11.81), and no low-score matches. Chemical synaptic transmission showed the highest score of 90.43 with 39 matched genes (see Table 5). No common genes were found for the top 10 GO–biological processes, although DRD2 and DRD3 were the most represented among six of the top 10 GO–biological processes. In the GO–molecular function category, 312 genes of the 332 addictive behavior-associated genes were matched to 132 entities. There were 72 high-score matches (range: 70.67 to 13.31), 60 medium-score matches (range: 13.25 to 9.69), and no low-score matches. Protein binding categories, identical protein binding, protein serine/threonine/tyrosine kinase activity, enzyme binding, kinase activity, and protein kinase activity scored >40.0 (see Table 6). The “protein binding” entity (score: 70.67) ranked first in the high-scoring match list consisting of 272 genes of our total compiled list of 332 genes by far, representing the category with the largest total number of genes (n = 13,902 genes). No genes were found in common representing the top 10 GO–molecular function category listed in Table 6, but one gene (*EGFR*) was implicated in eight of the top 10 GO–molecular functions.

**TABLE 5:** Top 10 GO–biological processes associated with clinically relevant and known genes for addictive behavior.

GeneAnalytics score	GO–biological processes	Total number of genes identified in the GO–biological process from integrated databases	Number of matched addictive behavior-associated genes from the master list (%)	Number of matched genes from integrated databases (%)	Overlapping genes in the top three GO–biological processes
90.43	Chemical synaptic transmission	260	39/332 (11.75)	39/260 (15.0)	None
85.27	Signal transduction	2,201	98/332 (29.52)	98/2,201 (4.4)	
80.63	Positive regulation of transcription by RNA polymerase II	1,196	70/332 (21.08)	70/1,196 (5.8)	
65.03	Positive regulation of gene expression	537	43/332 (12.95)	43/537 (8.0)	
64.25	Positive regulation of cell population proliferation	545	43/332 (12.95)	43/545 (7.8)	
61.76	Response to xenobiotic stimulus	266	31/332 (9.34)	31/266 (11.6)	
61.17	Locomotory behavior	92	21/332 (6.33)	21/92 (22.8)	
57.61	Positive regulation of peptidyl-serine phosphorylation	90	20/332 (6.02)	20/90 (22.2)	
51.42	Response to ethanol	113	20/332 (6.02)	20/113 (17.6)	
51.00	Positive regulation of the ERK1 and ERK2 cascade	230	26/332 (7.83)	26/230 (11.3)	

**TABLE 6:** Top 10 GO–molecular functions associated with clinically relevant and known genes for addictive behavior.

GeneAnalytics score	GO–Molecular functions	Total number of genes identified in GO–Molecular functions from integrated databases	Number of matched addictive behavior-associated genes from the master list (%)	Number of matched genes from integrated databases (%)	The 18 overlapping genes in the top three GO–molecular functions
70.67	Protein binding	13,902	272/332 (81.9)	272/13,902 (1.9)	ALK, **AKT1** , BRAF, EPHB2, ERBB2, ERBB3, **EGFR** , FGFR2, IGF1R, INSR **MAPK1**, **MAPK3** , MET, MTOR, PLK1, PIK3CG, RAF1, and TBK1
58.43	Identical protein binding	1,711	73/332 (21.9)	73/1,711 (4.3)	
46.79	Protein serine/threonine/tyrosine kinase activity	447	33/332 (9.9)	33/447 (7.4)	
40.91	Enzyme binding	397	29/332 (8.7)	29/397 (7.3)	
40.27	Kinase activity	697	38/332 (11.4)	38/697 (5.4)	
40.20	Protein kinase activity	529	33/332 (9.9)	33/529 (6.2)	
39.25	Transmembrane receptor protein tyrosine kinase activity	55	13/332 (3.9)	13/55 (23.6)	
35.21	DNA-binding transcription factor activity	675	35/332 (10.5)	35/675 (5.2)	
34.84	Sequence-specific DNA binding	474	29/332 (8.7)	29/474 (6.1)	
32.93	Protein tyrosine kinase activity	136	16/332 (4.8)	16/136 (11.8)	

Colored genes represent those present in more than one analyzed category.

#### 3.2.5 Phenotypes

This category showed 242 of the 332 compiled genes matched to 378 phenotypes. There were 296 high-score matches (range: 98.13 to 13.31), of which only the top 10 phenotypes were reported. There were 82 medium-score matches (range: 13.25 to 11.79). The decreased body weight phenotype had the highest matching score of 98.13 with 75 matched genes, as seen in Table 7. The *BDNF* gene was found in common and was represented in eight of the top 10 high-scoring phenotypes.

**TABLE 7:** Top 10 phenotypes associated with clinically relevant and known genes for addictive behavior.

GeneAnalytics score	Phenotype	Total number of genes identified in phenotypes from integrated databases	Number of matched addictive behavior-associated genes from the master list (%)	Number of matched genes from integrated databases (%)	The 27 overlapping genes in the top three phenotypes
98.13	Decreased body weight	1,128	75/332 (22.5)	75/1,128 (6.6)	ABL1, ACHE, **ACTB** , AR, **AKT1** , BCL2, **BDNF** , BRAF, CHRNA3, CREBBP, DRD1, DRD2, **EGFR** , GGT1, GRM1, **IGF1** , KCNJ6, KMT2A, MYC, OCA2, RAF1, SLC18A2, SLC6A3, SNAP25, TP53, **TH0** , and TPH2
81.76	Premature death	822	59/332 (17.7)	59/822 (7.2)	
66.04	Decreased body size	732	50/332 (15.0)	50/732 (6.8)	
61.17	Hyperactivity	270	31/332 (9.3)	31/270 (11.4)	
58.72	Postnatal growth retardation	576	42/332 (12.6)	42/576 (7.3)	
55.94	Hypoactivity	308	31/332 (9.3)	31/308 (10.1)	
53.00	Increased body weight	195	25/332 (7.5)	25/195 (12.8)	
52.00	Complete embryonic lethality during organogenesis	532	38/332 (11.4)	38/532 (7.1)	
49.68	Impaired conditioned place preference behavior	23	12/332 (3.6)	12/23 (52.2)	
44.64	Increased circulating insulin level	146	20/332 (6.0)	20/146 (13.7)	

Colored genes represent those present in more than one analyzed category.

#### 3.2.6 Compounds

From our total number of 332 addictive behavior-associated genes, 274 genes matched to 1,985 compounds. The high-score matches (range: 166.10 to 13.39) for the top 10 compounds are shown in Table 8. There were no identified medium or low scores. Dopamine, glutamate, diethylstilbestrol (DES), serine, and D-tyrosine had the highest matching scores of 166.10, followed by isotretinoin with a score of 163.60. In addition, the *BDNF* and *CREB1* genes were found to represent all the top 10 high-scoring compounds.

**TABLE 8:** Top 10 compounds associated with clinically relevant and known genes for addictive behavior.

GeneAnalytics score	Compounds	Total number of genes identified in compounds from integrated databases	Number of matched addictive behavior-associated genes from the master list (%)	Number of matched genes from integrated databases (%)	The 29 overlapping genes in the top three compounds
166.10	Dopamine	466	88/332 (26.51)	88/466 (18.9)	ADRA2A, **AKT1** , **BDNF** , BRAF, COMT, **CREB1** , CRH, CYP2A6, GAL, GCG, GDNF, HERT, INSR, MAOA, MAOB, **MAPK3** , MTHFR, MMP9, NFE2L2, **NPY** , NR3C2, NTRK2, PDYN, PIK3CA, PNOC, TAC1, **TH** , and TSPO
166.10	Glutamate	813	94/332 (28.31)	94/813 (11.6)	
166.10	Diethylstilbestrol	970	108/332 (32.53)	108/970 (11.1)	
166.10	Serine	1,647	139/332 (41.87)	139/1,647 (8.4)	
166.10	D-Tyrosine	1,788	137/332 (41.27)	137/1,788 (7.7)	
163.60	Isotretinoin	995	93/332 (28.01)	93/995 (9.3)	
162.76	Cocaine	157	49/332 (14.76)	49/157 (31.2)	
152.51	Gamma-aminobutyric acid	249	54/332 (16.27)	54/249 (21.7)	
152.45	NMDA (N-methyl-D-aspartic acid)	294	57/332 (17.17)	57/294 (19.4)	
151.47	Nicotine	198	50/332 (15.06)	50/198 (25.3)	

Colored genes represent those present in more than one analyzed category.

Analysis of the top three GeneAnalytics integrated scores across the seven GeneAnalytics categories of clinically relevant and known genes associated with addictive behavior revealed overlapping genes within the categories. Specifically, 67 overlapping genes were identified in the top three tissues and cell types in brain regions, 18 overlapping genes were identified in the top three Disease categories, 12 overlapping genes were identified in the top three Superpaths/pathways, 18 overlapping genes were identified in the top three GO–molecular function categories, 27 overlapping genes were identified in the top three Phenotypes, and 29 overlapping genes were identified in the top three Compounds. Notably, no overlapping genes were identified in the GO–biological process category. Across all seven Gene Analytics categories, only one gene, *AKT1*, consistently overlapped in five of the categories, as highlighted in bold and colored red in Tables 3, 4, 6, 7, 8. The *CREB1* gene overlapped in four categories and is highlighted in bold and colored blue in Tables 2, 3, 4, 8. Other genes that overlapped among the three categories were *ACTB* (Tables 2, 3, 7), *BDNF* (Tables 2, 7, 8), *IGF1* (Tables 2, 3, 7), *MAPK1* (Tables 2, 4, 6), *MAPK3* (Tables 4, 6, 8), *NPY* (Tables 2, 3, 8), *TH* (Tables 2, 7, 8), and *EGFR* (Tables 4, 6, 7), as highlighted in bold and colored gold.

### 3.3 STRING web-based integrated database and analysis

**FIGURE 1:**
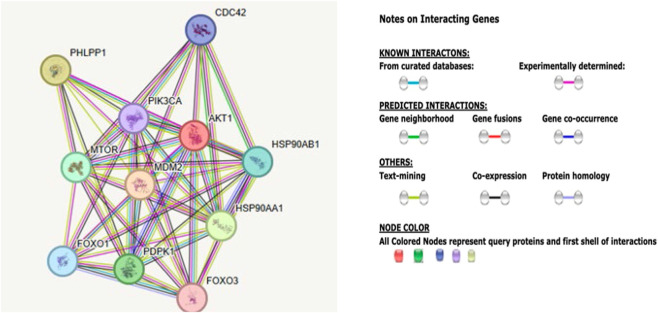
STRING *AKT1* protein interaction. The interactive network of the *AKT1* gene is shown along with functional associations involving 10 associated protein nodes representing the first-tier analysis and 49 edges with color coding, as illustrated and further described by Szklarczyk et al. (2023). Network nodes represent proteins with splice isoforms or post-translational modifications collapsed into each node for all proteins produced by a single protein-coding gene. Edges represent specific and meaningful protein–protein interactions, such as proteins that jointly contribute to a shared function. Different colored lines and nodes represent the known and predicted interactions and proteins analyzed in the network associated with this gene.

We selected the STRING web-based program and its analytical approach that studies one gene at a time to capture protein–protein interaction networks and functions associated with the single gene and its encoded protein. The most often identified gene that overlapped among the GeneAnalytics identified categories was *AKT1*, which was consistently present in five of the seven categories. No other gene showed this level of involvement. Hence, we analyzed the protein–protein interaction network, biological processes, molecular functions, pathways, and disease–gene associations related to *AKT1* using the methodology described in the STRING web-based program and database (https://string-db.org). Figure 1 represents the shared protein–protein interaction network centered around *AKT1* and its encoded protein.

AKT1 is a protein kinase specifically classified as a serine/threonine kinase that plays a crucial role in regulating cell growth, survival, metabolism, and angiogenesis. Protein phosphorylation is a fundamental process for the regulation of cellular function requiring the coordinated action of both protein kinases and phosphates that control the levels of phosphorylation; hence, it involves the activation of specific targeted signal transduction pathways with cAMP and protein kinase C (www.OMIM.org). The searchable first-tier STRING analysis for *AKT1* identified 10 interactive protein nodes, each representing all proteins produced by the corresponding gene and encoded protein, including isoforms, and 49 edges that indicate both direct and predicted functional as well as physical protein–protein associations with multiple signaling functional enrichments, as shown in Figure 1. The protein symbol and description of the function of each of the 10 proteins associated with *AKT1* are shown in Table 9.

**TABLE 9:** STRING: protein or gene symbols and descriptions of the top 10 associated proteins with AKT1*.

Protein symbol	Description
MDM2	E3 ubiquitin-protein ligase Mdm2, an E3 ubiquitin-protein ligase that mediates ubiquitination of p53/TP53 and inhibits p53/TP53- and p73/TP73-mediated cell-cycle arrest and apoptosis. Acts as an ubiquitin ligase E3 toward itself and ARRB1. Promotes proteasome-dependent ubiquitin-independent degradation of retinoblastoma RB1 protein and inhibits DAXX-mediated apoptosis. Encodes a nuclear-localized E3 ubiquitin ligase
PHLPP1	PH domain leucine-rich repeat-containing protein phosphatase is a serine/threonine phosphatase that regulates AKT and PKC signaling. Mediates dephosphorylation of members of the AGC Ser/Thr protein kinase family, specifically acts on “Ser-473” of AKT2 and AKT3, “Ser-660” of PRKCB, and “Ser-657” of PRKCA. Isoform 2 mainly regulates Akt signaling in hippocampal neurons, influencing cell survival and apoptosis. Encodes a member of the serine/threonine phosphatase family
HSP90AA1	Heat shock protein HSP90-alpha is a molecular chaperone that promotes the maturation, structural maintenance, and proper regulation of specific target proteins involved in cell-cycle control and signal transduction. Its ATPase activity drives conformational changes that activate client proteins. Interacts with co-chaperones that regulate its substrate binding, ATPase activity, and chaperone function
PDPK1	3-Phosphoinositide-dependent protein kinase 1is a serine/threonine kinase that phosphorylates and activates the AGC family of protein kinases. Its targets include protein kinase B (PKB/AKT1, PKB/AKT2, and PKB/AKT3), p70 ribosomal protein S6 kinase (RPS6KB1), p90 ribosomal protein S6 kinase (RPS6KA1, RPS6KA2, and RPS6KA3), cyclic AMP-dependent protein kinase (PRKACA), protein kinase C (PRKCD and PRKCZ), serum and glucocorticoid-inducible kinase (SGK1, SGK2, and SGK3), p21-activated kinase-1 (PAK1), and protein kinase PKN (PKN1 and PKN2)
MTOR	Serine/threonine kinase that regulates cellular metabolism, growth, and survival in response to hormones, nutrients, and stress. mTOR controls the phosphorylation of ∼800 proteins and functions in two complexes, mTORC1 and mTORC2. Activated mTORC1 promotes protein synthesis by phosphorylating key regulators of mRNA translation and ribosome synthesis
HSP90AB1	Heat shock protein HSP90-beta is a molecular chaperone that stabilizes and regulates target proteins involved in cell-cycle control and signal transduction. Its ATPase-driven cycle induces conformational changes in client proteins, enabling their activation. It interacts with various co-chaperones that modulate its substrate recognition and chaperone activity
FOXO1	Forkhead box protein O1 is a transcription factor regulated by insulin signaling that maintains metabolic and redox homeostasis. It binds IRE and DBE DNA elements, with the activity suppressed by insulin. FOXO1 controls osteoblast function, bone mass, and skeletal regulation of glucose metabolism, acting with ATF4 to modulate osteocalcin expression
CDC42	Cell division control protein 42 is a plasma membrane-associated small GTPase that switches between active (GTP-bound) and inactive (GDP-bound) states to regulate diverse cellular responses. It is involved in epithelial cell polarization and regulates spindle attachment during mitosis, cell migration, and filopodia formation in neurons
PIK3CA	Phosphatidylinositol 4,5-bisphosphate 3-kinase catalytic subunit alpha is a PI3K enzyme that generates PIP3 from phosphoinositide, recruiting PH domain proteins such as AKT1 and PDPK1. This activates the signaling pathways controlling cell growth, survival, proliferation, and motility
FOXO3	Forkhead box protein O3 (FOXO3) is a transcription factor that binds 5′-[AG]TAAA[TC]A-3′ DNA motifs to regulate apoptosis and autophagy. During starvation, it activates the autophagy genes in the skeletal muscle, promoting protein degradation, and induces apoptosis when the survival signals are absent

* STRING database (www.string-db.org); GeneCards database (www.genecards.org). The 10 protein nodes are arranged by their predicted functional analytical order that is key to carry out the function of AKT1.

The predicted functions of the top 10 proteins listed in Table 9 highlight the central role for AKT1 in the PI3K/AKT/mTOR signaling pathway, integrating extracellular growth signals to promote proliferation and inhibit apoptosis. Among the 10 protein interactions identified, MTOR, HSP90AA1, and PIK3CA overlapped with the genes listed as clinically relevant and involved in addictive behavior in Table 1. In addition, when extending the search to a third-tier analysis, comparing 30 associated proteins with AKT1 showed three additional proteins (PIK3CB, PIK3CD, and PIK3CG), which also overlapped with the addiction-associated genes.

The six proteins/genes that were found to interact with AKT1 and are present in our list of clinically relevant and known genes for addictive behavior (as identified by the GeneAnalytics program) were distributed across the seven GeneAnalytics categories, as observed when analyzing our 332 genes. We found that HSP90AA1, a heat shock protein, was present in five categories, excluding Tissues and Cells and Phenotypes. MTOR was present in six categories, excluding the Tissues and Cells category. PIK3CA was present in all seven categories. PIK3CB was present in six categories, excluding Tissues and Cells, and PIK3CD was present in five categories, excluding Tissues and Cells and Phenotypes. Finally, PIK3CG was present in four categories, excluding Tissues and Cells, GO–Biological Processes, and Phenotypes.

The top 10 significantly associated proteins and their genes with the predicted functional pattern for AKT1 are listed in Table 9. Among these, MDM2 is an E3 ubiquitin-protein ligase that mediates ubiquitination of p53/TP53. It is phosphorylated by AKT1, whereas PHLPP1 is a serine/threonine phosphatase that regulates AKT and PKC signaling and mediates dephosphorylation, specifically acting on “Ser-473” of AKT2 and AKT3. HSP90AA1 and HSP90 AB1 are both molecular chaperones that are involved in the maturation, structural maintenance, and proper regulation of specific target proteins involved in cell-cycle control and signal transduction. PDPK1 and MTOR control phosphorylation and activation. FOXO1 and FOXO3 are transcription factors, while FOXO1 is regulated by insulin signaling, which maintains metabolic and redox homeostasis and controls osteoblast function, bone mass, and skeletal regulation of glucose metabolism, while FOXO3 binds to 5′-[AG]TAAA[TC]A-3′ DNA motifs to regulate apoptosis and autophagy. CDC42 is a cell division control protein that is a plasma membrane-associated small GTPase that regulates diverse cellular responses. PIK3CA is a PI3K enzyme that produces PIP3 from phosphoinositide, which recruits PH domain proteins such as AKT1 and PDPK1. This initiates signaling pathways that regulate cell growth, survival, proliferation, and motility. The STRING database predicted protein functions with the top associated proteins of AKT1, which are described in Table 10, including biological processes, molecular functions, cell components, KEGG and Reactome pathways, and disease associations.

**TABLE 10:** STRING predicted functions for AKT1*.

Biological process	CIN^a^	Strength^b^	Signal^c^	FDR^d^
Anoikis (GO:0043276)	3 of 12	2.65	2.48	2.57e-05
Positive regulation of lamellipodium assembly (GO:0010592)	4 of 29	2.39	2.92	2.40e-06
Response to osmotic stress (GO:0006970)	4 of 78	1.96	2.15	3.35e-05
Cellular response to oxygen levels (GO:0071453)	5 of 152	1.77	2.21	8.39e-06
Regulation of protein kinase B signaling (GO:0051896)	5 of 166	1.73	2.11	1.22e-05
Molecular function	CIN	Strength	Signal	FDR
dATP binding (GO:0032564)	2 of 2	3.25	1.42	0.0028
CTP binding (GO:0002135)	2 of 2	3.25	1.42	0.0028
Sulfonylurea receptor binding (GO:0017098)	2 of 3	3.38	1.41	0.0028
UTP binding (GO:0002134)	2 of 3	3.38	1.41	0.0028
Nitric-oxide synthase regulator activity (GO:0030235)	3 of 8	2.83	2.19	0.00010
Cellular component	CIN	Strength	Signal	FDR
Dendritic growth cone (GO:0044294)	2 of 9	2.6	0.99	0.0159
Sperm plasma membrane (GO:0097524)	2 of 15	2.38	0.95	0.0182
Axon growth cone (GO:0044295)	2 of 25	2.16	0.83	0.0288
Endocytic vesicle (GO:0030139)	4 of 338	1.33	0.77	0.0182
Mitochondrion (GO:0005739)	6 of 1681	0.81	0.47	0.0316
KEGG pathway	CIN	Strength	Signal	FDR
Longevity regulating pathway-multiple species (hsa04213)	5 of 61	2.17	3.87	6.13e-09
Prostate cancer (hsa05215)	8 of 97	2.17	5.82	2.70e-14
Thyroid hormone signaling pathway (hsa04919)	6 of 120	1.95	3.66	2.29e-09
AMPK signaling pathway (hsa04152)	6 of 120	1.95	3.66	2.29e-09
Fox0 signaling pathway (hsa04068)	6 of 126	1.93	3.62	2.29e-09
Reactome pathway	CIN	Strength	Signal	FDR
Constitutive signaling by AKT1 E17K in cancer (HSA-5674400)	6 of 26	2.62	5.61	1.17e-11
CD28 dependent P13K/Akt signaling (HSA-389357)	4 of 22	2.51	3.6	1.27e-07
CD28 co-stimulation (HSA-389356)	5 of 33	2.43	4.29	3.42e-09
P13K/AKT signaling in cancer (HSA-2219528)	7 of 105	2.08	4.46	5.85e-11
VEGFA–VEGFR2 pathway (HSA-4420097)	6 of 96	2.05	3.79	3.42e-09
Disease–gene association	CIN	Strength	Signal	FDR
*Proteus* syndrome (DOID:13482)	2 of 3	3.08	1.05	0.0130
Cowden syndrome (DOID:6457)	2 of 10	2.55	0.96	0.0182
PTEN hamartoma tumor syndrome (DOID:0080191)	2 of 10	2.55	0.96	0.0182
Head and neck cancer (DOID:11934)	2 of 12	2.47	0.95	0.0182
Urinary system cancer (DOID:3996)	3 of 89	1.78	0.89	0.0182

* STRING (string-db.org) predicted functions for AKT1.

^a^ CIN (count-in-network) indicates how many proteins in the network are annotated with a particular term and how many proteins in total (in the network and in the background) have this term assigned to this variable per category (biological process, molecular function, etc.).

^b^ Log10 (observed/expected) or strength. This measure describes how large the enrichment effect is with the ratio between i) the number of proteins in the network that are annotated with a term and ii) the number of proteins expected to be annotated with this term in a random network of the same size.

^c^ Signal is defined as a weighted harmonic mean between the observed/expected ratio and log (FDR). FDR tends to emphasize larger terms due to their potential for achieving lower *p*-values, while the observed/expected ratio highlights smaller terms, which have a high foreground-to-background ratio but cannot achieve low FDR values due to their size.

^d^ FDR is a statistical measure that describes the significance of enrichment. *P*-values corrected for multiple testing within each category are shown.

### 3.4 Overlapping genes in addiction and alcoholism

In our study on genes contributing to addiction, we found that 29 genes overlapped with the 337 genes reported in alcoholism or alcohol use disorder previously (Manzardo et al., 2015). The genes in common for both addiction and alcoholism indicate that these conditions share common biological processes, pathways, or functions, particularly in the brain, and the involvement of several organ systems having different tasks (see Table 11). Some overlapping genes, like *ADH1B* and *ALDH2*, are involved in alcohol metabolism and may influence how the body processes alcohol. Other genes, such as *DRD2*, *ANKK1*, and *NR4A2*, affect dopamine signaling, which is central to the reward system and for motivation and craving. Opioid-related genes, such as *OPRM1*, *OPRD1*, and *POMC* (a major obesity-related gene), also influence the brain’s reward and stress responses. In addition, several genes were involved with neuroplasticity and growth with overlap, including *BDNF*, *FGFR2*, *IGF1*, and *IGF1R*, indicating structural and functional changes in the brain that may contribute to addiction vulnerability. Two classic obesity-related genes, namely, *POMC* and *NPY*, directly involve the control of eating, including hyperphagia, while other genes are involved with neurological and brain functions, including *BDNF*, *DRD2*, *NRXN2*, *OPRD1*, *OPRM1*, *CHRNA3*, and *CHRNA5*, which contribute to addiction. Furthermore, separate genes were found to regulate growth and growth factors, including *FGFR2*, *FN1*, *IGF1*, and *IGF1R*, along with *SLC6A*, a recognized neurotransmitter gene. Together, these genes demonstrate that addictive behavior and alcoholism do share overlapping biological processes and genetic mechanisms, affecting the reward pathways, stress response, neurotransmission, and brain plasticity. This added genetic information could impact the direction of research for therapeutic interventions.

**TABLE 11:** Twenty-nine clinically relevant genes that are common to addictive behavior and alcoholism.

Gene symbol	Gene name	Chromosome location
*ADH1B*	Alcohol dehydrogenase 1B (class I), beta polypeptide	4q23
*ALDH2*	Aldehyde dehydrogenase 2 family	12q24.12
*ALK*	ALK receptor tyrosine kinase	2p23.2-p23.1
*ANKK1*	Ankyrin repeat and kinase domain-containing 1	11q23.2
*ATP6V1B2*	ATPase H+ transporting, lysosomal 56/58-KD, V1 subunit B2	8p21.3
*BDNF*	Brain-derived neurotrophic factor	11p14.1
*CALM2*	Calmodulin 2	2p21
*CHRM2*	Cholinergic receptor, muscarinic, 2	7q33
*CHRNA3*	Cholinergic receptor, neuronal nicotinic, alpha polypeptide 3	15q25.1
*CHRNA5*	Cholinergic receptor, neuronal nicotinic, alpha polypeptide 5	15q25.1
*CSMD1*	CUB, and sushi multiple domains 1	8p23.2
*DRD2*	Dopamine receptor D2	11q23.2
*FGFR2*	Fibroblast growth factor receptor 2	10q26.13
*FN1*	Fibronectin 1	2q35
*GABBR1*	GABA gamma-aminobutyric acid receptor 1	6p22.1
*GABRA2*	GABA gamma-aminobutyric acid receptor, alpha-2	4p12
*GSN*	Gelsolin	9q33.2
*IGF1*	Insulin-like growth factor 1	12q23.2
*IGF1R*	Insulin-like growth factor 1 receptor	15q26.3
*NPY*	Neuropeptide Y	7p15.3
*NR4A2*	Nuclear receptor subfamily 4, group A, member 2	2q24.1
*NRXN3*	Neurexin 3	14q24.3-q31.1
*OPRD1*	Opioid receptor, delta-1	1p35.3
*OPRM1*	Opioid receptor, mu-1	6q25.2
*PEA15*	Proliferation and apoptosis adapter protein 15	1q23.2
*PEBP1*	Phosphatidylethanolamine-binding protein 1	12q24.23
*POMC*	Proopiomelanocortin	2p23.3
*SCD*	Stearoyl-CoA desaturase	10q24.31
*SLC6A*	Solute carrier family 6 (neurotransmitter transporter, serotonin), member 4	17q11.2

## 4 Discussion

Addiction is often seen as a choice or a consequence of poor decisions. However, the literature shows the importance of genetics in addiction, as described in our study. We identified 332 addictive behavior-related genes and studied genes with protein interactions and molecular functions. The genes that were studied often affect signaling pathways and neurotransmitters, especially dopamine, serotonin, GABA, and glutamate involving reward response, impulse control, and emotional regulation. The key genes included *DRD2* involved in the brain reward pathways and *SLC6A3* that regulates dopamine uptake and is, in turn, associated with reduced dopamine receptor availability, leading to a higher risk for alcoholism, cocaine, opioid, and nicotine use (Volkow et al., 2010). The *OPRM1* gene and its protein play a role in binding opioids (natural and synthetic) to their receptors and influences individual sensitivity to opioids and is associated with an increased risk of opioid addiction (Zhang et al., 2005). The *CHRNA5/A3/B4* gene cluster has a strong association with nicotine dependence and heavy smoking behavior (Bierut et al., 2008). COMT is involved in the breakdown of dopamine, epinephrine, and norepinephrine, with the Val158Met polymorphism affecting dopamine levels in the prefrontal cortex, influencing cognitive control and substance use risk. In addition, *GABRA2* gene variants are linked to increased risk of alcohol dependence and behavioral disinhibition (Edenberg et al., 2004). In our study, we showed 29 clinically relevant genes that are common in addictive behavior and alcoholism, indicating an interaction between the two disorders that warrants more studies.

### 4.1 GeneAnalytics program, analysis, and identified factors

The GeneAnalytics program results were divided into seven specific categories, namely, Tissues and Cells, Diseases, Superpaths/pathways, GO–Biological Processes and GO–Molecular Functions, Phenotypes, and Compounds.

#### 4.1.1 Tissues and cells

The cerebellum was qualified as a high-match and the top anatomical brain compartment of the gene expression analysis in the Tissues and Cells category.

Addictions are considered brain disorders based on drug use and seeking behavior, regardless of the harmful consequences (Leonardi et al., 2015), and they are involved with functional changes to brain circuits for reward, stress, and self-control. Genetic factors, along with socioenvironmental factors (e.g., psychosocial), have been established as significant contributors to addiction and vulnerability (Nestler, 2013; Vassoler and Sadri-Vakili, 2014; Saunders et al., 2022). In addition, addictive drugs can produce long-lasting molecular and structural plasticity alterations in the corticostriatal-limbic system (Kalivas et al., 2005; Hyman et al., 2006). An increasing amount of data further indicate the involvement of the cerebellum in many of those brain functions that are affected in people with addiction (Miquel et al., 2009; Moulton et al., 2014; Wang and Lan, 2023).

The molecular and cellular actions of addictive drugs in the cerebellum involve long-term adaptative changes to the receptors, neurotransmitters, and intracellular signaling transduction pathways, leading to the reorganization of cerebellar micro-zones and, in turn, to functional networks where the cerebellum is an important nodal structure (Miquel et al., 2009; Moulton et al., 2014). Cerebellar structural alterations are also linked to addiction, as shown by Lacomba-Arnau and colleagues in 2025, and an interruption in the typical structural development in an affected lateral frontoparietal network, indicating an extended pattern of neuro-regulation within the cerebellar network in individuals with cocaine use disorder (Lacomba-Arnau et al., 2025).

In the Tissues and Cells category, high-matched genes occupied 3.4% of the total number of genes in the cerebellum. In the medium-matched genes occupied 4.9% of the total number of genes in the medulla oblongata, 2.8% of the total number of genes in the cerebral cortex, 5.5% of the total number of genes in the hypothalamus, and 5.2% of the total number of genes in the thalamus, respectively. The paraventricular thalamic nucleus (PVT) is also considered an important component of neural circuits underlying drug addiction (Martin-Fardon and Boutrel, 2012; James and Dayas, 2013; Matzeu et al., 2014; Kirouac, 2015). Anatomically and functionally, the PVT regulates drug-related behaviors by receiving extensive inputs from the brainstem and hypothalamus, which have a reciprocal connection with the limbic system. Neurons in the PVT are also recruited by drug exposure, as well as cues and contexts associated with drug consumption (Zhou and Zhu, 2019). Our studies further showed that the compiled list of 36 addictive behavior genes was found in common in all five brain tissue regions identified using the GeneAnalytics web-based program and databases.

#### 4.1.2 Diseases

In the Diseases gene category and analysis of addictive behavior, schizophrenia had the highest score and accounted for 17.8% of all matched genes. Substance use disorder is also particularly prevalent in the schizophrenic population (Selzer and Lieberman, 1993). Schizophrenic populations commonly use one or more substances, including nicotine, alcohol, cannabis, cocaine, and amphetamines (Mueser et al., 1990; Dixon et al., 1991; Cuffel, 1992; Zisook et al., 1992; Selzer and Lieberman, 1993). Substance use comorbidity in schizophrenia is often thought to be a form of self-medication, where individuals use substances to relieve symptoms of the disorder—such as positive and negative symptoms, cognitive deficits, or side effects from antipsychotic medications. Emerging evidence indicates that neuropathology found in schizophrenia may directly affect brain circuits involved in drug reward and reinforcement, thus increasing vulnerability to addiction. Abnormalities in the hippocampus and frontal cortex may also enhance drug reward effects and impair control over drug-seeking behavior. These effects are partly due to disrupted dopamine and glutamate signaling in the nucleus accumbens, leading to addiction-like neural and motivational changes—even without prior drug use. This implies that addictive behavior in schizophrenia may be a core symptom of the disorder that occurs alongside or independently of other symptoms (Chambers et al., 2001).

Other disease genes in addictive behaviors are also found in diabetes mellitus type 2 at 11.5%, nervous system disease at 10.7%, Alzheimer disease, familial, at 9.2%, body mass index quantitative trait locus at 11 9.0%, lung cancer at 5.9%, colorectal cancer at 5.7%, and breast cancer at 5.3%. The *MTOR* and *PTGS2* genes were found in all top 10 diseases in the Diseases category. Additionally, Pastor et al. (2020) reported an association between alcohol and recreational drugs with type 2 diabetes mellitus.

#### 4.1.3 Superpaths/Pathways

Superpaths/Pathways provide insights into broader cellular processes and networks using groups of genes based on their participation in shared biological pathways. As shown in our study, signal transduction, endometrial cancer, and GPCR downstream signaling were the top three pathways, along with *CREB1*, *MAPK1*, *MAPK3*, and *AKT1* genes being present in nine of the top 10 identified pathways.

Signal transduction plays a key role in addictive behavior by facilitating how the neuronal brain cells respond to drugs and reinforcement signals. Addiction alters normal signaling pathways, especially those involved in reward, learning, and memory, leading to long-lasting changes in brain function and behavior (Nestler, 2001; Zinsmaier et al., 2025). GPCRs also play a central role in mediating neurotransmitter signaling at chemical synapses, especially in the brain regions associated with reward and reinforcement, such as the nucleus accumbens and ventral tegmental area.

Neurotransmitters such as dopamine and endogenous opioids will bind to GPCRs, triggering intracellular cascades via G proteins that regulate secondary messengers including cAMP and calcium (Beaulieu and Gainetdinov, 2011). These signaling pathways activate kinases such as PKA and MAPK, which modulate synaptic plasticity by phosphorylating ion channels and transcription factors such as CREB, leading to changes in gene expression and neuronal excitability (Nestler, 2004; Kauer and Malenka, 2007; Zhang et al., 2022). Drugs that are abused also disrupt normal GPCR signaling by overstimulating or inhibiting these pathways, resulting in maladaptive synaptic modifications that underlie addictive behaviors such as tolerance, dependence, and craving (Lüscher and Malenka, 2011). Thus, alterations in GPCR downstream signaling at chemical synapses are fundamental to the neurobiological mechanisms of addiction. The *AKT1* gene encodes a serine/threonine kinase that plays a pivotal role in the dopamine signaling pathway, particularly downstream of dopamine D2 receptors. *AKT1* further modulates dopamine neurotransmission by regulating key downstream effectors such as glycogen synthase kinase-3 beta (GSK-3β), thus influencing neuronal responses to rewarding stimuli. This modulation affects synaptic plasticity and neuroadaptive changes in brain regions central to addiction, such as the nucleus accumbens and prefrontal cortex. Additionally, genetic variations in *AKT1* have been linked to altered susceptibility to addictive behaviors and substance-induced psychiatric conditions, highlighting its importance in addiction vulnerability (Beaulieu et al., 2005; Lai et al., 2006; Di Forti et al., 2012).

#### 4.1.4 GO–Biological Processes and GO–Molecular Functions

No gene was found to be common in representing the 10 GO–biological processes, but chemical synaptic transmission had the highest score, followed by signal transduction and positive regulation of transcription by RNA polymerase II. *DRD2* and *DRD3* were commonly represented among six of the 10 GO–biological processes. In the GO–Molecular Function category, protein binding had the highest score, with the *EGFR* gene implicated in eight of the top 10 GO–Molecular Functions. Repeated exposure to drugs that are abused alters chemical synaptic transmission (GO:0007268) in key reward-related brain regions such as the nucleus accumbens and prefrontal cortex. These changes initiate signal transduction pathways (GO:0007165) involving secondary messengers and kinase cascades, ultimately leading to positive regulation of gene expression (GO:0010628) via transcription factors (CREB, deltaFosB, and NF-κB), which stabilize long-term neuroadaptations. The mesolimbic dopamine system, consisting of the ventral tegmental area (VTA) and nucleus accumbens (NAc), as well as associated limbic structures are critical for neural adaptations that underline addiction (Nestler, 2001; Kauer and Malenka, 2007).

In GO–Molecular Function, protein binding has the highest score. Central to these processes are proteins that mediate protein binding (GO:0005515) and enzyme binding (GO:0019899), enabling the assembly of signaling complexes. Crucially, many of these proteins exhibit kinase activity (GO:0016301), including protein serine/threonine kinase activity (GO:0004674) and protein tyrosine kinase activity (GO:0004713), which modulate transcription factors such as CREB (Carlezon et al., 2005). Phosphorylated CREB then enhances the expression of downstream targets such as BDNF, thus reinforcing synaptic plasticity and creating persistent changes in neuronal circuitry that underline drug craving, relapse vulnerability, and the compulsive nature of addictive behaviors (Shaywitz and Greenberg, 1999; Nestler, 2013).

#### 4.1.5 Phenotypes

Among the top 10 phenotypes reported in our study related to addictive behavior, decreased body weight had the highest score with 6.6% matching genes from integrated databases. Some of the genes found in our analysis are also linked to decreased or altered body weight, such as *CRH*, *HTR2C*, *FTO*, *GHSR*, *NPY*, *AGRP*, *BDNF*, *GLP1R*, *DRD2*, and *INSR*. These genes can play important roles in appetite regulation and body weight control, and changes in their expression or function can contribute to decreased body weight. Drugs such as cocaine and amphetamines are also linked to decreased body weight (Ersche et al., 2013).

BDNF plays a key role in neuroplasticity and neuronal survival. Polymorphisms in the *BDNF* gene, particularly the common Val66Met (rs6265) variant, can influence stress response and reward-related learning, thereby impacting an individual’s susceptibility to addiction (Cheng et al., 2005). BDNF is involved in the regulation of energy balance and appetite. Decreased BDNF expression is linked to increased food intake and obesity, while enhanced BDNF signaling can reduce weight (Gray et al., 2006).

#### 4.1.6 Compounds

Dopamine and glutamate had the highest matching scores of 18.9% and 11.6%, respectively, matching genes from the integrated databases. Interestingly, the *BDNF* and *CREB1* genes were found in all of the top 10 high-scoring compounds. As discussed, *BDNF* plays a major role in neurogenesis, neuroplasticity, cognition, and modulation of major neurotransmitter systems such as the dopaminergic, glutamatergic, and serotonergic system (Tyler et al., 2002; Gratacos et al., 2007). The *BDNF* Val66Met polymorphism is a significant genetic factor that modulates brain function related to addiction and influences how the brain responds to reward, stress, and drug-related cues, making it a vulnerability or resilience factor depending on the context (Notaras et al., 2015).

CREB1 is a transcription factor activated primarily by phosphorylation at Ser133 through dopamine and other neurotransmitter signaling pathways (Carlezon et al., 2005; Wang et al., 2018). Upon activation, CREB1 binds to cAMP response elements (CRE) in the DNA, promoting the transcription of downstream target genes such as *BDNF* and dynorphin (Shaywitz and Greenberg, 1999). Elevated CREB1 activity upregulates BDNF expression, which in turn enhances synaptic plasticity and neural remodeling within reward-related brain regions such as the hippocampus and VTA–NAc (Tao et al., 1998). This CREB1–BDNF signaling cascade underlies neuroadaptations that contribute to the persistence of addictive behaviors by creating a feed-forward loop that reinforces the addiction circuitry (Bali and Kenny, 2019).

### 4.2 STRING *in silico* web-based analysis

We identified 332 genes for addiction or addictive behavior from the literature and other sources and used the GeneAnalytics platform to study these genes. We found that the *AKT1* gene was the most consistently involved gene in the studied categories. Hence, the *AKT1* gene was chosen for analysis using the STRING web-based program to study protein–protein interactions, biological processes, molecular functions, pathways, and disease–gene associations. The results showed that the *AKT1* gene and its encoded protein plays an important role in multiple molecular networks involving protein phosphorylation impacting neurological functions, cellular components, and signaling pathways with functions, underlying addictive behavior. Notably, components of the PI3K–AKT–mTOR pathway have been repeatedly implicated in drug-induced neuroplasticity, axon growth, dopaminergic signaling, and behavioral sensitization (Russo et al., 2010; Li et al., 2022; Zhang et al., 2022).

We further examined the protein–protein interaction network of AKT1 using the STRING analytical approach and identified 30 proteins interacting with AKT1 across three interaction tiers. Several genes overlapped with our list of genes for addictive behavior, such as *HSP90AA1*, *HSP90AB1*, *MDM2*, *PHLPP1*, *CDC42*, *mTOR*, and *PIK3CA* in the first-tier analysis of 10 protein–protein interactions and *PIK3CB*, *PIK3CD*, and *PIK3CG* in the third-tier analysis with 30 protein–protein interactions. These genes are key regulators of the PI3K–AKT signaling axis, which is critically involved in synaptic plasticity and the long-term neuroadaptations induced by addictive substances (Franco-García et al., 2023; Sharma et al., 2025). *HSP90AA1* and *HSP90AB1* encode a molecular chaperone required for the conformational maturation and stabilization of multiple signaling proteins, and functional HSP90 is needed for the stability of Akt in the complex. Loss of HSP90 function destabilizes AKT1 within this complex (Basso et al., 2002). The mTOR is found in two functionally distinct complexes, mTORC1 and mTORC2, that crucially control long-term synaptic efficacy and memory storage. Dysregulation of the two complexes has been implicated in a variety of neurodevelopmental and neuropsychiatric disorders, including drug addiction (Costa-Mittioli and Monteggia, 2013). The mTORC2 complex directly phosphorylates AKT1 at Ser473, which is a modification that is required for full AKT activation; mTORC2 also associates with ribosomes, supporting co-translational AKT maturation (Oh et al., 2010). Hence, the MTOR in the AKT1 network reinforces connection to addiction biology. In addition, MDM2 is an E3 ubiquitin-protein ligase for regulation of cell-cycle arrest and apoptosis, while both PHLPP1 and CDC42 proteins are involved with neuron formation, neuro-development (hippocampus), and function, as noted in our study.

The *PIK3CA*, *PIK3CB*, *PIK3CD*, and *PIK3CG* genes encode catalytic subunits of class I PI3Ks. Three catalytic isoforms p110α, p110β, and p110δ are encoded by the genes *PIK3CA*, *PIK3CB*, and *PIK3CD*, respectively, and a single class IB catalytic isoform p110γ is encoded by the gene *PIK3CG*. These kinases activate AKT1 by generating phosphatidylinositol-(3,4,5)-trisphosphate (PIP3) at the plasma membrane (Madsen and Toker, 2023; Shaw et al., 2025). PI3K signaling contributes to addiction by AKT1-dependent neuroadaptation in the reward circuitry (Szumlinski et al., 2019). Together, these findings place AKT1 as a highly interconnected signaling center within addiction-relevant molecular networks as well as an association with the *PTEN* gene, which is a tumor-suppressor gene implicated in addiction, autism, and cancer (Genovese and Butler, 2025). Our study may stimulate more research as important genes and encoded proteins are involved in playing a wide-ranging role of activation across several molecular mechanisms contributing to addiction in humans.

Potential limitations of our study were raised, including the need to undertake additional statistical analysis and testing beyond that performed by the two established web-based analytical programs with built-in statistical approaches to formally evaluate whether the observed proportions of addiction-related genes across the biological categories differ significantly from those expected by chance. As the GeneAnalytics web-based platform incorporates its own embedded statistical framework, we did not perform independent analyses to directly compare the proportions reported in Tables 2–8 and the number of matched addictive behavior-associated genes from the master list in relation to the number of matched genes from the integrated databases with the total number of genes identified per biological category. For example, 523 genes were identified and studied using the GeneAnalytics program for schizophrenia and compared with the number of genes in common with addiction (N = 332), having 93 genes in common in the Diseases category in relation to other diseases (see Table 3). An approach could be considered in the future but is beyond the scope of this study.

The advances in research and new information are useful for clinical practice, as seen in Table 4, where pharmacodynamics and involved genes (N = 18) represented 83% of matched genes (15/18) in the integrated database but only 4.5% of addiction-related genes (15 of 332), which appears to be low or a discrepancy. Pharmacogenetics and its use in clinical care for treatment purposes is on the rise and, thus, is a relatively new field in medical care and management. More work is needed to identify other involved genes impacting the selection and use of existing and new drugs for treatment. Furthermore, the number of genes and our understanding of involvement of new and existing genes playing a role in the seven biological categories described in this study should be continued as these areas of research are not consistent; with new genetic factors reported overtime, incorporating such statistical validation could strengthen the interpretation of category-specific enrichment and represent an important direction for future work.

## 5 Conclusion

Treatment is often necessary to manage addiction, which is a complex, chronic disease that requires a multifaceted therapeutic approach, often involving medications, therapy, and long-term support to achieve and maintain recovery. Our approach identified 332 genes related to addiction from the literature and other sources and examined their encoded proteins. We used two separate computational biological web-based programs to search and analyze protein–protein interactions, pathways, and molecular functions and found the most relevant addiction-associated genes as AKT1*, CREB1, MAPK1, MAP3,* and *BDNF* and signaling pathways (e.g., PI3/AKT/mTOR) affecting the reward system. We describe how the components interact across multiple biological levels that may stimulate research on therapeutic strategies. Our study examined associations at the tissue and cell level as well as diseases, functions, cellular processes, phenotypes, and compounds, emphasizing that tissue-specific differences in gene expression patterns and protein production should be considered in research investigations. Neurological or psychiatric disorders such as addiction are brain disorders and often require brain tissue analysis, making genetics and protein-based research more challenging. Twenty-nine of the 332 genes associated with addiction were also reported in alcoholism or alcohol use disorder. Together, these findings enhance our understanding of the complex biological processes underlying addiction and may increase the knowledge and insights into a very complex disease process. This research may open new avenues for causation, diagnosis, therapeutic approaches, and intervention that are important for those affected with addiction.

## Data Availability

The original contributions presented in the study are included in the article/supplementary material; further inquiries can be directed to the corresponding author.
